# Implications of peritoneal cancer index distribution on patients undergoing cytoreductive surgery and hyperthermic intraperitoneal chemotherapy

**DOI:** 10.1515/pp-2021-0150

**Published:** 2022-04-26

**Authors:** Jolene Si Min Wong, Grace Hwei Ching Tan, Sabrina Hui Xian Cheok, Chin-Ann Johnny Ong, Claramae Shulyn Chia, Melissa Ching Ching Teo

**Affiliations:** Department of Sarcoma, Peritoneal and Rare Tumours (SPRinT), Division of Surgery and Surgical Oncology, National Cancer Centre Singapore, Singapore, Singapore; Department of Sarcoma, Peritoneal and Rare Tumours (SPRinT), Division of Surgery and Surgical Oncology, Singapore General Hospital, Singapore, Singapore; SingHealth Duke-NUS Surgery Academic Medical Program, Duke-NUS Medical School, Singapore, Singapore; SingHealth Duke-NUS Oncology Academic Medical Program, Duke-NUS Medical School, Singapore, Singapore; Laboratory of Applied Human Genetics, Division of Medical Sciences, National Cancer Centre Singapore, Singapore, Singapore; Institute of Molecular and Cell Biology, A*STAR Research Entities, Singapore, Singapore

**Keywords:** cytoreductive surgery, hyperthermic intra-peritoneal chemotherapy, peritoneal cancer index

## Abstract

**Objectives:**

Peritoneal cancer index (PCI) score is a common prognostication tool in peritoneal metastases (PM). We hypothesize that the distribution of PCI score and involvement of specific regions affects survival and morbidity outcomes.

**Methods:**

Data was collected from a prospective database of patients who underwent CRS and HIPEC for PM at the National Cancer Centre Singapore. We evaluate the relationship between PCI, PCI distribution, and survival and morbidity outcomes.

**Results:**

One hundred and fifty-two patients underwent CRS and HIPEC with a median PCI score of nine (range 0–31). Median overall survival (OS) and progression free survival (PFS) were 43 and 17 months, respectively. Region six (pelvis) was most commonly involved and had the highest frequency of heavy disease burden. Presence of PM in the lower abdomen, flanks, and small bowel were associated with poorer OS (p=0.01, 0.03, <0.001) and PFS (p=0.04, 0.02, <0.001). Involvement of porta hepatitis predicted poorer OS but not PFS (p=0.03). Involvement of the gastric antrum resulted in higher rates of postoperative complications.

**Conclusions:**

The pattern of PCI distribution may be associated with varying survival and morbidity outcomes.

## Introduction

Since its introduction in the 1990s, Sugarbaker’s peritoneal cancer index (PCI) has been widely adopted, providing a means for standardized reporting of the extent of peritoneal involvement during cytoreductive surgery and hyperthermic intraperitoneal chemotherapy (cytoreductive surgery [CRS] and hyperthermic intraperitoneal chemotherapy [HIPEC]) for various peritoneal surface based malignancies [1, 2]. It is a score that takes into account both the peritoneal implant size as well as its distribution in 13 abdominopelvic regions. When compared with other intraoperative assessment tools, such as, the Gilly’s peritoneal carcinomatosis staging, Japanese ‘S, N, P, H’ system, or the Dutch proposed simplified PCI and ‘7 Region Count’, Sugarbaker’s PCI was found to be superior in terms of its prognostic value, ability to predict complete cytoreduction, and reproducibility by experts worldwide [2], [3], [4].

The importance of accurate quantification of peritoneal metastases (PM) cannot be understated, especially in colorectal and ovarian primaries, where extensive disease often precludes effective complete CRS and HIPEC and is associated with early failure and poor survival outcomes [5], [6], [7]. In colorectal PM, a linear relationship has been found between PCI and survival outcomes [8]. Similarly, in ovarian PM, PCI has been shown to predict the likelihood of complete CRS and has a prognostic impact [8, 9].

As the PCI scoring system was designed with an intention to provide a reliable and reproducible method to assess the extent of peritoneal disease and with potential prognostic implications, it does not contain specific information on regions of involvement that may also be of interest to peritoneal surgeons. For example, the involvement of certain anatomical structures like the porta hepatitis or caudate which may be associated with greater ‘technical’ difficulties is not elaborated by the PCI score alone. Based on radiological studies, it has been shown that intraperitoneal fluid movement and hence tumor implantation distribution follow certain patterns [10]. Dominant regions of involvement include the omentum and cul-de-sac while the small bowel and its mesentery have reduced incidence of disease due to peristalsis [11]. As such, we hypothesized that the involvement of specific PCI regions, as well as anatomically critical structures may have a differential impact on surgical morbidity and survival outcomes, in patients with PM undergoing CRS and HIPEC.

We aim to evaluate: (1) the pattern of distribution of PCI score in patients undergoing CRS and HIPEC and (2) determine the relationship between PCI distribution and survival and morbidity outcomes.

## Subjects and methods

### Patient selection and data

This is a retrospective cohort study performed in a single tertiary institution. Data was retrieved from a prospectively maintained database of patients treated with CRS and HIPEC for PM between January 2001 and January 2018. Patients with PM secondary to colorectal, ovarian, and other histological subtypes (mesothelioma and primary peritoneal malignancies) were included. Patients with PMP, multicystic mesothelioma, and incomplete CRS were excluded.

This study was conducted with approval of the Centralized Institutional Review Board of Singapore Health Services. It adheres to the STROBE (Strengthening the Reporting of Observational Studies in Epidemiology) guidelines for observational studies.

### Definitions

Intraoperatively, the PCI score was tabulated and used to quantify the extent of peritoneal disease. Based on this scoring system, the abdominal cavity was divided into 13 regions and the disease burden in each of these regions were defined by lesion size (LS) scores [1].

Based on the team’s clinical experience with the management of CRS and HIPEC cases, five critical areas of involvement were defined as: (1) the porta hepatitis, (2) caudate lobe of liver, (3) gastric antrum, (4) spleen, and (5) diaphragmatic surfaces. The involvement of these areas has been found to be associated with added surgical morbidity and may have a prognostic impact after CRS and HIPEC [12], [13], [14], [15]. As such, their involvement were recorded and analysed separately for each patient. The completeness of cytoreduction (CC) score was utilized to measure the amount of residual disease [16], with CC-0/1 considered as optimal cytoreduction.

All intra- and postoperative complications were recorded and graded based on the Clavien-Dindo classification [17], with grade three and above complications classified as major events.

Overall survival (OS) was defined as time in months between CRS and HIPEC to date of last follow-up or death. Progression free survival (PFS) was defined as the time in months from the date of CRS and HIPEC to the date of detection of recurrent disease.

### CRS and HIPEC and follow-up

The CRS and HIPEC procedure performed at our institution was as previously described [18, 19] and involves the removal of all macroscopic peritoneal disease to achieve optimal cytoreduction with the subsequent administration of HIPEC. A closed technique for HIPEC was adopted with 4 L of peritoneal dialysis solution at 41–42 °C over a duration of 60 min. Mitomycin C was administered for colorectal PM while cisplatin was given for non-gastrointestinal PM. A Belmont^®^ hyperthermia pump was used during the study duration to deliver the intraperitoneal chemotherapy agent via a single inflow catheter and drainage was via four intra-abdominal drains.

Postoperatively, patients were transferred to the surgical intensive care unit (SICU) or high-dependency unit for monitoring. During the follow-up, patients were reviewed at three monthly intervals during which a full physical examination and markers (Ca 125, CEA and Ca19-9) were taken. A computed tomography (CT) scan of the chest-abdomen and pelvis was performed 6 monthly for the first 2 years post-CRS and HIPEC and then yearly thereafter or when clinically indicated [18]. Details of recurrences, if any, were recorded.

### Statistical analysis

The relationship between overall PCI score, lesion size score distribution in each PCI region, and the involvement of any of the five critical organs with postoperative morbidity and survival outcomes were analysed. Wilcoxon rank-sum test was used if the distributions were skewed for numeric variables used. Categorical variables were evaluated using the Pearson chi-square or Fisher’s exact tests. Survival functions were estimated using Kaplan–Meier method and log-rank test was used to evaluate the differences between the two groups. Multivariate Cox proportional hazards (PH) models were built for OS and PFS, and multivariate logistic regression models were built for occurrence of high-grade complications using a forward stepwise variable selection model. PH assumption was verified based on Schoenfeld residuals. A two-sided p-value of <0.05 was taken as significant. All analyses were performed in PASW Statistics 18.0.2.

## Results

### Patient and tumor characteristics

A total of 205 patients underwent CRS and HIPEC during the study duration with 152 meeting the inclusion criteria. 61 (40%) patients had PM secondary to colorectal primary, 57 (37.5%) ovarian, and 34 (22.5%) others (20 primary peritoneal, 10 mesotheliomas, and four small bowel). Due to the small numbers in ‘other’ histological subtypes, these patients were analysed collectively. Patients with missing data were excluded from the analysis. All patients and tumor characteristics are described in Table 1.

**Table 1:** Demographics and clinical characteristics of CRS and HIPEC patients.

	CRS and HIPEC patients (n=152)
**Patient characteristics**	
Age, years, mean (range)	52.5 (24–76)
Gender	
** **Male	32 (21%)
** **Female	120 (79%)
Race	
** **Chinese	119 (78%)
** **Others	33 (22%)
ECOG status	
** **0/1	120 (79%)
** **2	32 (21%)
**Tumor characteristics**	
** **Colorectal	61 (40%)
** **Ovarian	57 (37.5%)
** **Others	34 (22.5%)
**Intraoperative**	
PCI score, median (range)	9 (0–25)
** **PCI colorectal PM	7 (1–21)
** **PCI ovarian	9 (0–15)
** **PCI primary peritoneal	13 (0–25)
PCI score	
** **≤15	119 (78%)
** **>15	33 (22%)
CC-score	
** **CC0	136 (90%)
** **CC1	16 (10%)

CRS and HIPEC, cytoreductive surgery and hyperthermic intra-peritoneal chemotherapy; ECOG, Eastern Cooperative Oncology Group; PCI, peritoneal cancer index; PM, peritoneal metastases; CC, completeness of cytoreduction.

### PCI and distribution

Median PCI score was nine (range 0–25). The central pelvis, represented by region six, was the most commonly involved region with the highest incidence of heavy disease burden (i.e. lesion size, LS 3, >5 cm or confluent peritoneal disease).

**Figure 1:**
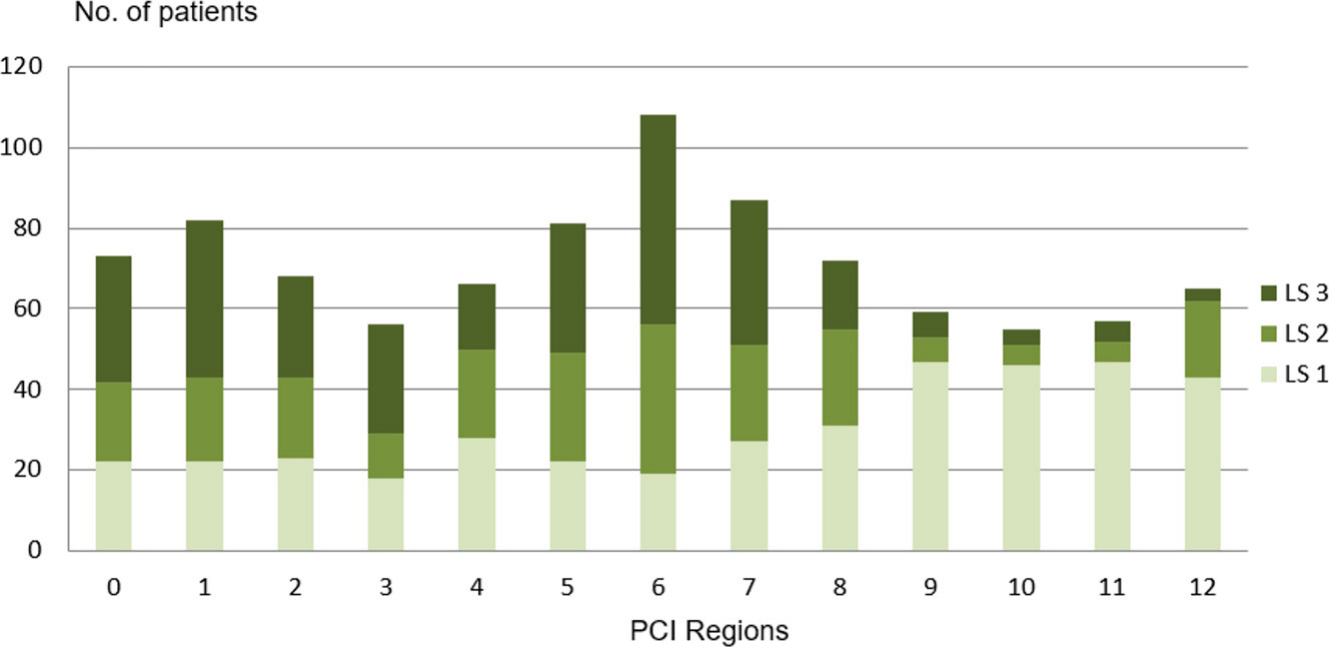
Pattern of PCI distribution.

The lower abdomino-pelvic region, represented by regions 5–7, appeared to be more frequently involved than the mid (regions 0, 4, 8) and upper (regions 1–3) areas. The small bowel (represented by regions 9–12) when involved had predominantly low volume disease, i.e. LS 1, aggregate tumor size <0.5 cm. Figure 1 depicts the frequency of peritoneal disease in each of the 13 defined PCI regions.

When comparing colorectal, ovarian, and ‘other’ PM, there was no significant difference in the distribution of peritoneal disease in the 13 defined PCI regions (p=0.112).

### Morbidity outcomes

Median CRS and HIPEC duration was 480 min (range 185–960 min) with an average blood loss of 1 L (range 100 mL to 8 L). Average length of hospitalization stay was 14 days (range 7–120 days) with five patients requiring ICU postoperatively and median ICU stay duration of one day.

Postoperative complications occurred in 31.5% (n=48) patients with 10% (n=15) suffering grade three and above major events. Only the involvement of the gastric antrum requiring gastric resection was significantly associated with the occurrence of postoperative morbidity. Diaphragmatic involvement, disease at the porta hepatitis, spleen, and caudate were not associated with increased morbidity rates.

### Survival and recurrence outcomes

**Figure 2:**
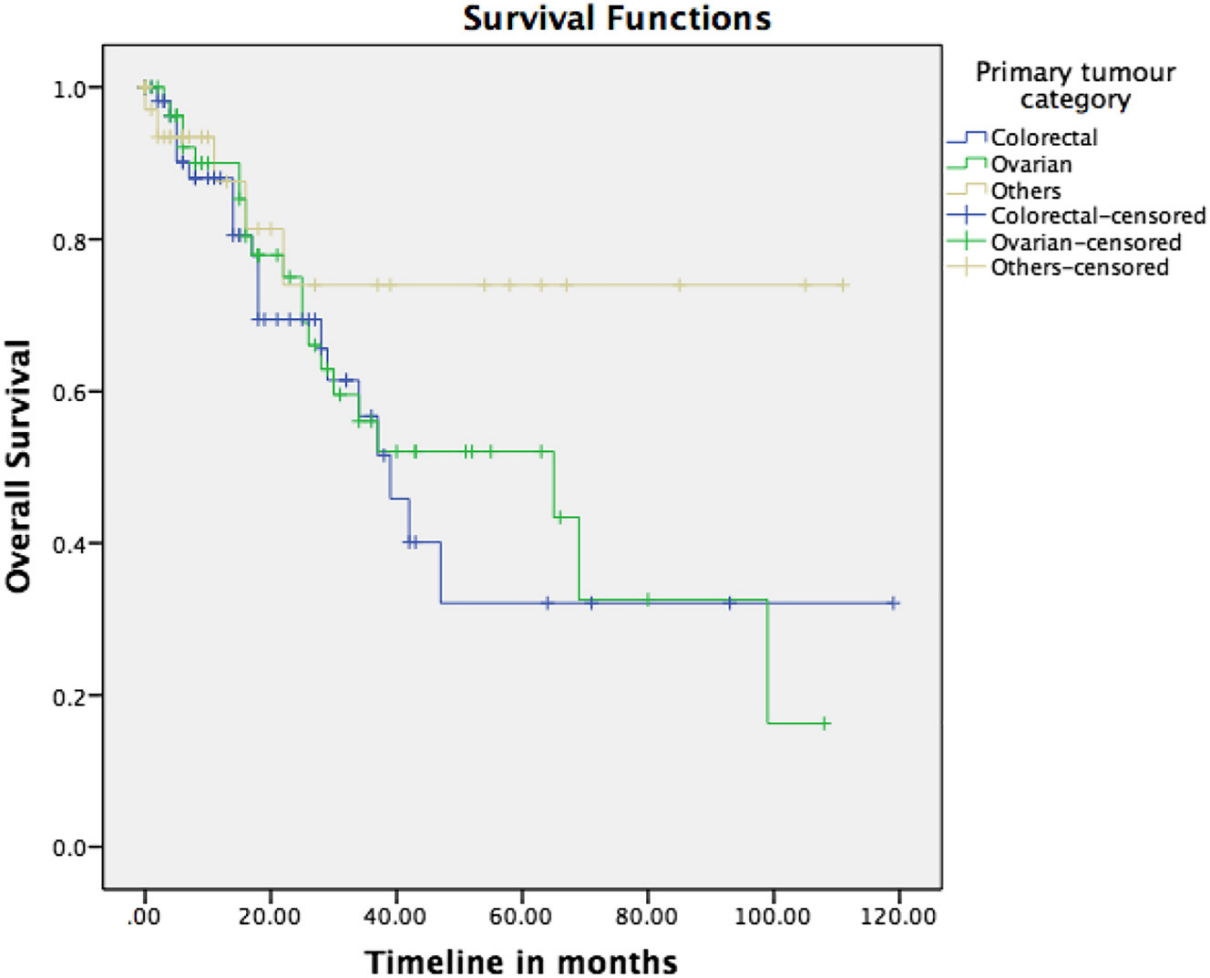
Overall survival (OS) of patients after CRS and HIPEC.

**Figure 3:**
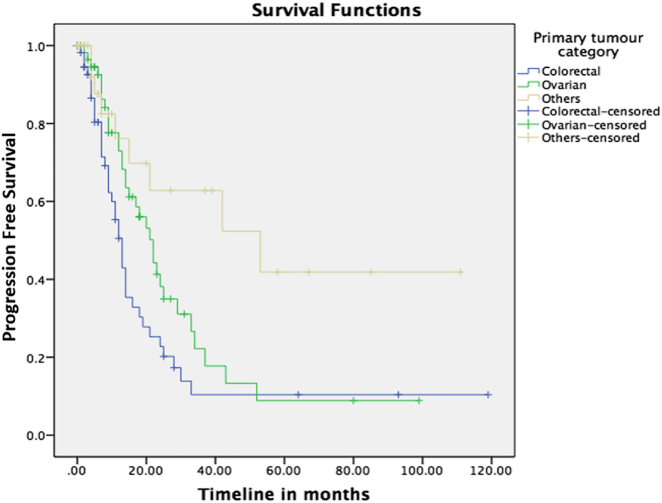
Progression free survival (PFS) of patients after CRS and HIPEC.

Overall median OS and PFS was 43 and 17 months, respectively, in all CRS and HIPEC patients. 1, 3, and 5-year OS and PFS for the various primaries are illustrated in Table 2 and Figures 2 and 3. While there was no difference seen in OS, patients with non-colorectal PM have a significantly improved PFS outcome when compared with the colorectal group (p=0.001).

**Table 2:** Overall survival (OS) and progression free survival (PFS) patients after CRS and HIPEC.

Overall survival						
	No. of events/No. of patient	Median, (95% CI)	1 year rate, % (95% CI)	3 year rate, % (95% CI)	5 year rate, % (95% CI)	p-Value
All patients	46/152	43 (2.9–83)	88.8%	59.6%	49.8%	
Primary						0.101
Colorectal	20/61	39 (29.2–48.0)	88.1%	56.7%	32.1%	
Ovarian	21/57	65 (13–116.2)	90%	56.1%	52.1%	
Others	5/34	40 (25–60.5)	87.6%	74%	NA	

**Progression free survival**						

All patients	80/152	17 (12.5–21.4)	64.4%	24.8%	16.1%	
Primary						0.001
Colorectal	38/61	13 (10.6–15.3)	50.5%	10.4%	NA	
Ovarian	33/57	22 (16.2–27.0)	72.9%	22.2%	8.9%	
Others	9/34	53 (9–96.0)	76%	62.8%	41.9%	

### The relationship between PCI distribution and survival outcomes

**Figure 4:**
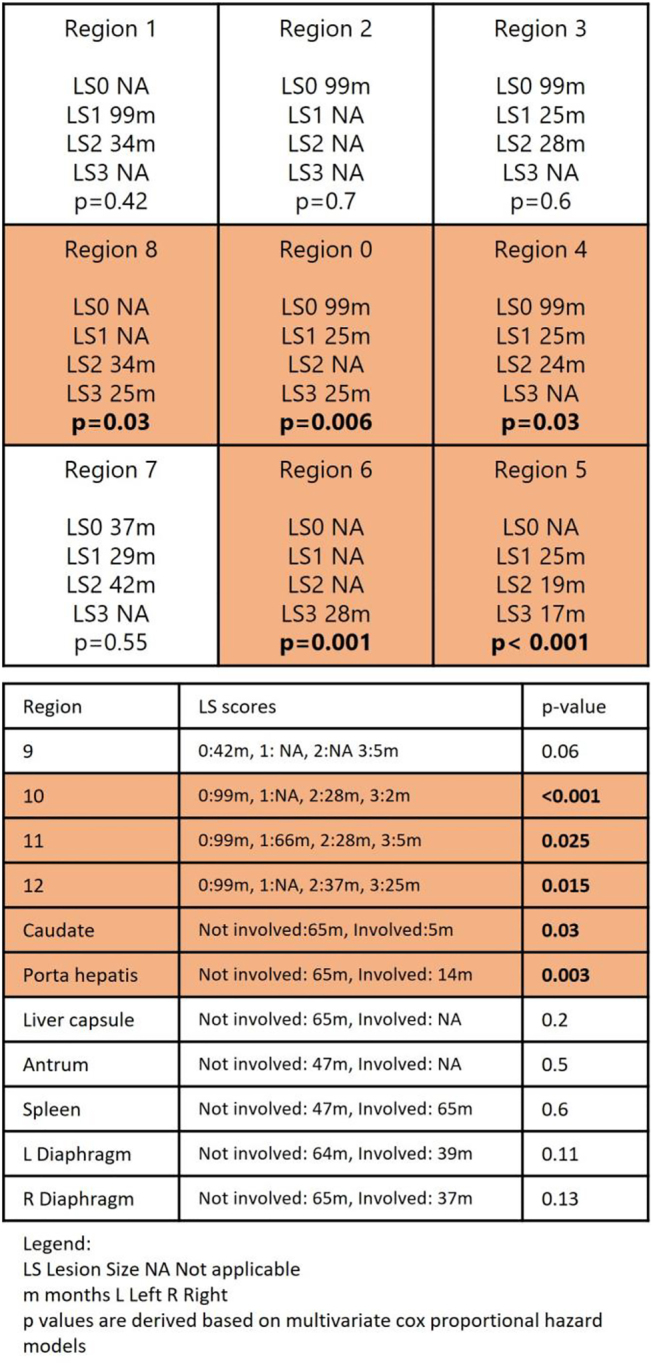
PCI distribution/lesion size (LS) score and overall survival (months).

**Figure 5:**
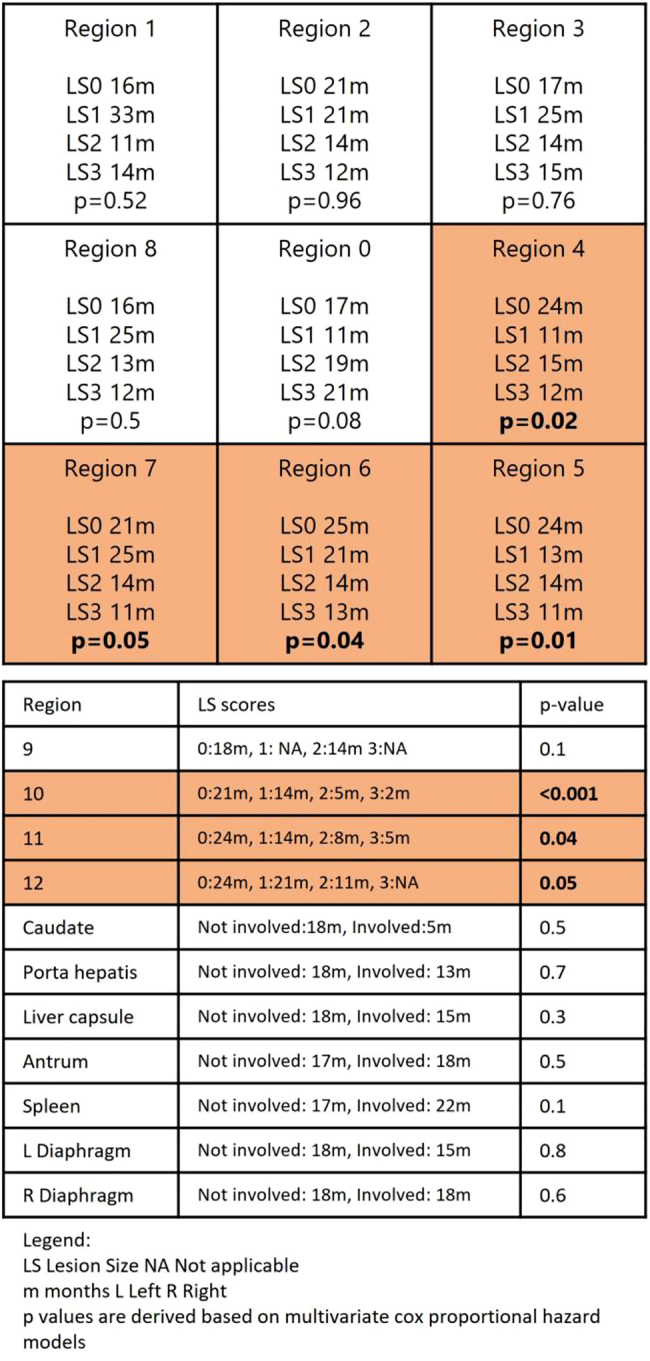
PCI distribution/lesion size (LS) score and progression free survival (months).

In all CRS and HIPEC patients, peritoneal implants in the pelvis, flanks (regions 4–8), and small bowel (regions 10–12) were significantly associated with poorer OS and PFS. Involvement of the porta hepatitis predicted poorer OS but not PFS (Figures 4 and 5).

In the colorectal subset, in addition to involvement of the pelvis, flanks, and small bowel, peritoneal disease at the caudate, porta, and bilateral diaphragms also resulted in significant reduction in OS outcomes (p=0.007, 0.001, 0.004, respectively). In contrast, for patients with ovarian and other subtypes, the involvement of the upper abdomen ‘crucial ’organs did not result in a difference in survival nor recurrence outcomes.

## Discussion

The ‘tumor cell entrapment’ hypothesis coined by Sugarbaker seeks to elucidate the rationale behind the development of PM after primary surgery [20]. It proposes that intraoperative seeding of tumor cells during surgical dissection, manipulation, and its subsequent embedment on exposed peritonised surfaces results in the inevitable development of PM [21,22]. Therefore, it predicts that regions most consistently involved by PM are likely (1) in close proximity with the primary tumor or (2) traumatized regions as a result of previous surgical dissection. In ovarian cancer, it was found that the vaginal cuff and abdominal incision sites free of cancer after hysterectomy but at high risk for tumor cell entrapment were disproportionately common sites for cancer found at reoperation [23]. In addition, the pattern of peritoneal fluid resorption, transport, and other factors such as bowel peristalsis, respiration, and gravity also have an impact on the distribution of tumor implants; with increased risks of occurrence of metastases in areas such as subphrenic region, diaphragm, and pelvis [20]. In our study, the pelvis (region six) was the most commonly involved and boosts the highest frequency of heavy disease burden during CRS and HIPEC, corresponding well with the above described fluid distribution phenomenon.

While the prognostic and predictive value of PCI has been widely validated, our study represents the first attempt to evaluate the pattern of PM as scored by the PCI and its association with survival and morbidity outcomes. In the preoperative context, the most common pattern of peritoneal involvement seen on CT imaging in colorectal cancer was in the pericolonic and pelvis regions in the form of scattered nodules [24]. In ovarian PM, predominant involvement of the pelvis, followed by the greater omentum and small bowel mesentery were found [25]. Intraoperatively, Spiliotis et al. reported that the small bowel PCI score was a likely predictor of survival in patients with colorectal PM after CRS and HIPEC [26]. The small bowel and its mesentery, with their constant peristalsis, have often a reduced incidence of peritoneal disease [11]. As such, it is not surprising that, when the small bowel is extensively involved, a ‘more advanced’ disease state is likely and this can be associated with poorer survival outcomes – a theory supported by the findings of this study where small bowel involvement was significantly associated with reduced OS. Similarly, while the upper abdomen and central regions had higher incidences of disease, their involvement was less likely to be associated with a poor OS than when the flanks were involved.

Disease affecting ‘crucial’ organs such as the porta hepatis, caudate, liver, stomach, spleen, and diaphragms are not specifically accounted for in the PCI scoring system. While we hypothesize that their involvement might be associated with survival outcomes, only disease encasing the portal triad was found to be a significant predictor. This is not surprising, as the completeness of CRS likely played a greater prognostic role than PCI [4]. As all our patients received complete CRS, the involvement of critical organs in the context of a CC-0/1 resection may have limited oncological impact. However, it is known that gastrectomy as part of the CRS and HIPEC procedure in lower gastrointestinal malignancies has been associated with increased rates of re-operation and prolonged hospitalisation stay [27], [28], [29]. Similar findings was seen in our patient cohort as well, elucidating to the fact that involvement of ‘crucial’ organs are more likely to impact morbidity outcomes rather than survival.

Our study, though shedding light on new information on the pattern distribution of PCI and its impact, does have limitations. The collection of PCI data was retrospective in nature with possible inconsistencies in its recording in the operative notes by the various surgeons. In addition, in this study, colorectal, ovarian, and ‘other’ PM were included – given their differing disease biologies, the true impact of PCI distribution and survival may be obscured.

In conclusion, the pelvis is the most commonly and heavily involved site of PM in our patients undergoing CRS and HIPEC. Involvement of the lower abdomen–pelvic regions, flanks, and small bowel portends a poorer prognosis when compared with upper abdominal disease. When complete CRS and HIPEC is performed, the involvement of crucial organs has more impact on morbidity rather than survival outcomes.
